# Effects of Long-Term Enclosed Environment on Human Health Based on the Analysis of Salivary Microbiota and Cytokines

**DOI:** 10.1128/spectrum.00254-22

**Published:** 2022-03-07

**Authors:** Zikai Hao, Yinzhen Zhu, Yuming Fu, Jianlou Yang, Chen Meng, Chen Dong, Hong Liu

**Affiliations:** a Beijing Advanced Innovation Centre for Biomedical Engineering, Beihang University, Beijing, China; b Institute of Environmental Biology and Life Support Technology, School of Biological Science and Medical Engineering, Beihang University, Beijing, China; c International Joint Research Center of Aerospace Biotechnology & Medical Engineering, Beihang University, Beijing, China; d State Key Laboratory of Software Development Environment, School of Computer Science and Engineering, Beihang University, Beijing, China; e Laboratory of Sport Nutrition and Intelligent Cooking, Shandong Sport University, Jinan, China; Lerner Research Institute

**Keywords:** long-term enclosed environment, salivary microbiota, salivary cytokines, immunity

## Abstract

The long-term exposure to enclosed environments may lead to chronic stress in crewmembers and affect their physical and mental state. Salivary microbiome and biomarkers of immune function are increasingly used in human health research. The “Lunar Palace 365” project, which was a 370-day, multicrew, enclosed experiment carried out in a ground-based bioregenerative life support system platform named Lunar Palace 1 (LP1). We investigated the temporal dynamics of the salivary microbiota and cytokines in the third phase of the “Lunar Palace 365” experiment, including 1 month before entering LP1 and 1 month after leaving Lp1. Results reveal no regular temporal change pattern in these parameters (highly abundant phyla and genera) during the experiment. Although the crewmembers’ oral microbiota temporally changed, it recovered quickly after the study subjects left the enclosed environment. The levels of IL-6, IL-10, and TNF-α in crewmembers’ saliva decreased after leaving the normal environment for the enclosed environment, indicating that their oral inflammatory response level was reduced. There were significant individual differences in crewmembers’ salivary microbiota, however, the shared living space reduced these differences. Moreover, air microbiota might have also played a significant role in reducing the individual differences. In summary, the enclosed environment did not result in persistent changes in human salivary microbiota and oral immunity. This study provides some insights for studying the effect of enclosed controlled environments on human immunity and microbiome.

**IMPORTANCE** Long-term exposure to space environments may influence the human microbiome, the human immune system, and the intricate balance between the two, causing impaired immunity and increased disease susceptibility. It was previously believed that the main potential factors of long-term spaceflight on human health were microgravity and radiation. However, the effects of long-term enclosed environments on human health were unclear. Bioregenerative life support systems (BLSS) is a good experimental model for studying the effects of enclosed environments on human systemic microbiota and immune disorders. We monitored the microbiota and cytokines in the saliva of crewmembers before they entered BLSS, during their stay in BLSS, and after leaving BLSS. The results indicated long-term closed environment will not cause persistent changes in human salivary microbiota and immunity.

## INTRODUCTION

Long-term space missions and the prospect of future life in space motivate research on the health of astronauts (1). Studies have shown that extreme pressure environments such as microgravity, cosmic radiation, and enclosed environments influence the human microbiome, immune system, and the intricate balance between the two, causing impaired immunity and increased disease susceptibility (2–5). The first two factors have been widely studied, whereas the impact of environmental isolation on astronauts’ health and microbiome has only been investigated more recently (6).

Blood and urine samples are generally considered to be the best way to assess physical health. However, collecting blood or urine samples may be difficult or impossible in some study designs. Collecting saliva samples is increasingly becoming a substitute for blood samples (7). The oral cavity is a gateway for pathogens and toxicants that enter the human body. Oral microbiota plays a vital role in maintaining both oral and general health (8). Oral mucosal epithelial cells and dendritic cells can distinguish between symbiotic and pathogenic microbiota by pattern recognition receptors, such as Toll-like receptors, and mediate immune inflammatory responses to intercept potential invading pathogens or show immune tolerance to systemic microbiota (9, 10). In addition, some symbiotic microbes can be pathogenic when they overgrow or are mislocated. Disturbance of the oral microbiota not only causes various oral infections, but is also associated with digestive disorders, cardiovascular diseases, diabetes, and rheumatoid arthritis (11, 12). Salivary inflammatory biomarkers are also believed to reflect an interaction between systemic and local immune activity as well as oral microbiome (13, 14). Recently, studies have found that long-term space flights can cause changes in the astronauts’ oral microbiota (15), causing oral and systemic inflammatory diseases. In Skylab missions, moderate increases were observed in the in-flight occurrence of dental plaque, calculus and gingival inflammation, and elevated counts of oral Streptococcus, *Neisseria*, *Lactobacillus*, and enteric bacilli were detected after flight (16). A number of other studies have reported that astronauts may suffer from conjunctivitis, upper respiratory tract infections, viral gastroenteritis, rhinitis, and skin infections (17). In the past, it was considered that the main underlying factors of long-term spaceflight on human health were microgravity and radiation (18). However, the effects of long-term enclosed environments in the isolation cabins on human health were unclear. Therefore, research on the changes in astronauts’ salivary microbiome and inflammatory biomarkers during space missions can provide some insights for assessing the oral and general health status of astronauts.

BLSS are artificial ecosystems with highly regenerated materials (food, oxygen, and water) that are essential for human life support (19, 20). BLSS can provide sustainable life support in a closed artificial ecosystem and represents the future direction of space stations and planetary bases (21). Lunar Palace 1 (LP1) is one of the most advanced BLSS, which offers a unique experimental model for studying the effects of space capsules on human systemic microbiota and immune disorders. LP1 has the following characteristics: (i) it is an enclosed experimental system with no material exchange with the outside world, which is beneficial to maintain a stable environment for the microbiome in the system; (ii) weekly disinfection effectively prevents microbial reproduction and provides an enclosed controlled environment for crewmembers (22); (iii) crewmembers work according to a fixed schedule and maintain a good psychological situation and oral health/hygiene (23); and (iv) the diet in BLSS remains basically constant, which can reduce the impact of dietary changes on salivary microbiota (24). We speculate that long-term living in this enclosed controlled environment will reduce crewmembers’ exposure of environmental microbiome, which may cause salivary microbial changes and oral inflammatory response disorders. On the other hand, when crewmembers return to the normal environment, the increased exposure to other microbes may cause the alteration of salivary microbiota and increase oral inflammatory response.

In order to prove our hypothesis, we investigated the effect of enclosed environment on the crewmembers’ symbiotic microbes and immune system in BLSS. We monitored the microbiota and cytokines in the saliva of crewmembers before they entered LP1, during their stay in LP1, and after leaving PL1. We also discuss the impact of decreased environmental microbial exposure on the salivary microbiota and cytokines.

## RESULTS

### Stability of salivary microbiota and individual differences.

**FIG 1:**
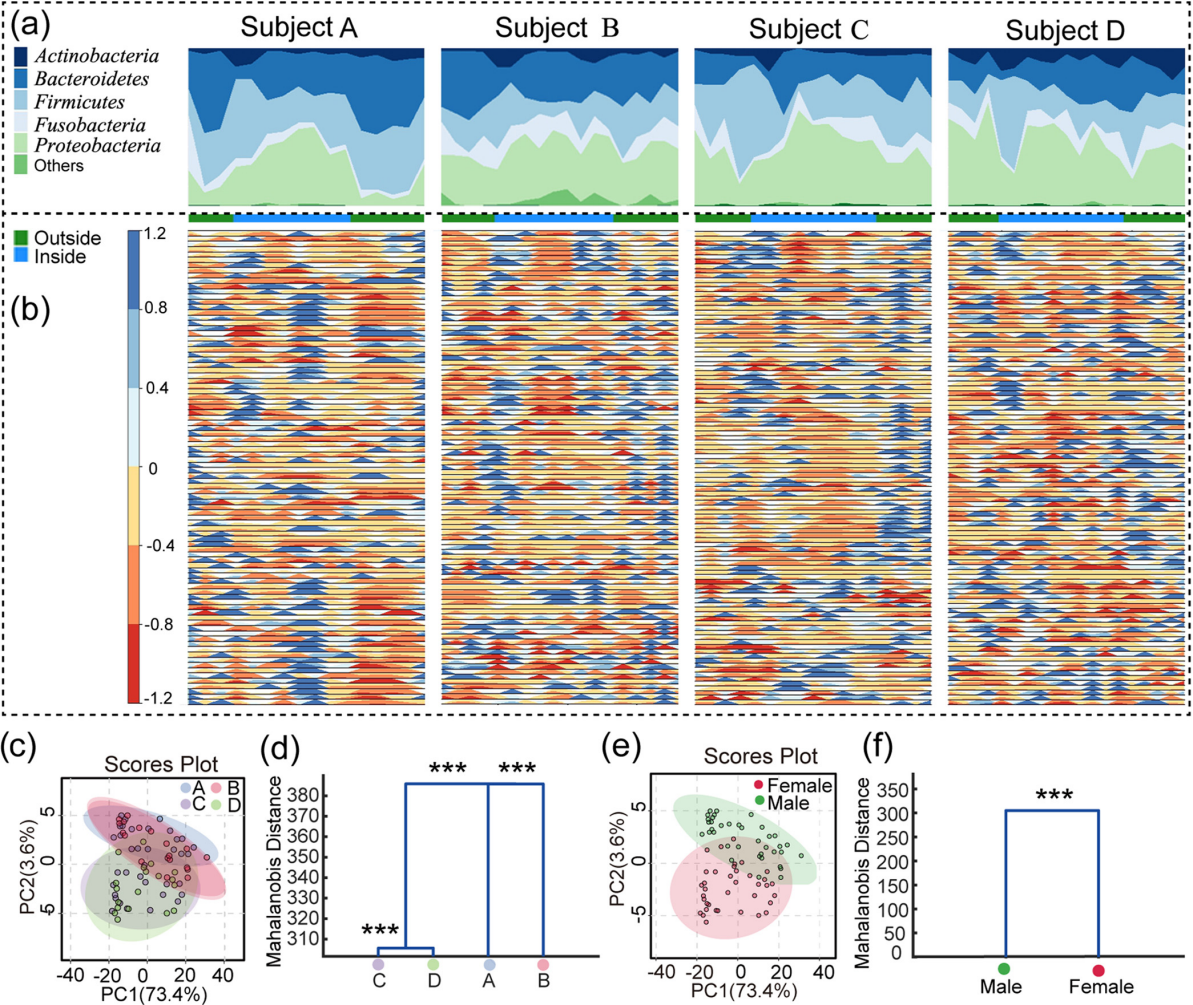
Dynamics and individual differences of the salivary microbiota during the whole experiment. (a) Percentage stacked graph showing phylum fractional abundances over time. Each ribbon represents a phylum, whose width is proportional to the phylum relative abundance at a given time point (the ribbons at the bottom of each plot indicate whether the crewmembers were outside or inside the LP1). (b) Horizon graphs of the relative abundance variation of highly abundant OTUs over time. The time series were mean-centered and the curves were divided into colored bands, whose width were the mean absolute deviation, which were then overlaid with the negative values mirrored upwards. Warm and cool colors indicate relative abundance below or above the mean, respectively; the darker the color, the smaller/greater the OTU abundance. The squares on the vertical axis were colored as in (a). For the list of highly abundant OTUs, please see Table S1. (c, e) The PCA scores the plots based on OTUs of the samples at the subject and gender, respectively. (d, f) MANOVA analysis of the different groups. The statistical significance of separation among the groups was assessed by MANOVA test based on Mahalanobis distances using the first 25 PCs of PCA at the subject and gender. The clusters were computed by applying the single linkage method to the matrix of Mahalanobis distances between the group means. *, *P < *0.05; **, *P < *0.01; ***, *P < *0.001.

The salivary microbiota of the four crewmembers (subject A to D) was tracked over time during the third phase of the “Lunar Palace 365” experiment. A total of 70 saliva samples were collected. Each sample was measured by 16S rRNA gene Illumina HiSeq sequencing (V3 to V4 region), and a total of 3,888,658 pairs of high-quality sequence reads were obtained. After splicing and filtering, a total of 3,312,409 clean tags were generated (minimum of all samples, 17,811; mean of all samples, 33,820). The dynamics of each crewmember’s oral microbial community were reconstructed over time according to the normalization strategy (25). The most abundant phyla encountered were Actinobacteria, Bacteroidetes, Firmicutes, Fusobacteria, Proteobacteria, and Spirochaetes, accounting for more than 97% in most samples (Fig. 1a). We selected the operational taxonomic units (OTUs) that existed at more than half of the time points as core OTUs for the dynamics analysis. We obtained 97, 104, 111, and 108 core OTUs in subject A, B, C, and D, respectively (Table S1). As shown in Fig. 1a, b and Fig. S1, these dynamic changes revealed that the changes of salivary microbiota between different individuals are also different over time. We visualized the differences in salivary microbiome between individuals and genders using principal-component analysis (PCA). Interestingly, individual and gender differences were differentiated on the PCA score plots in Fig. 1c and e. We further performed multivariate analysis of variance (MANOVA) to statistically compare individual and gender differences between the groups. Hierarchical clustering was generated based on Mahalanobis distances as a result of MANOVA. The MANOVA analysis showed that there were significant differences (*P*<0.001) between each individual and genders (Fig. 1d, f). We also found the individual differences at the different experimental phases (Fig. S2).

We adopted the autocorrelation function (ACF) to analyze the temporal dynamics of highly abundant phyla and genera during the entire experiment (26, 27). Our autocorrelation analyses showed that highly abundant phyla (Fig. S3) and genera (Fig. S4) had no significant autocorrelation, and they were stationary stochastic processes, suggesting that the crewmembers’ salivary microbiota exhibited no reliable changes with time during the experiment. Furthermore, the Mann-Kendall trend test (28, 29) was used to analyze whether there was a changing trend in the continuous time period from the normal environment into LP1, and the results showed that the trend was mostly not significant (*P*>0.05, Table S2).

### The shared living space reduced individual differences in the salivary microbiota.

**FIG 2:**
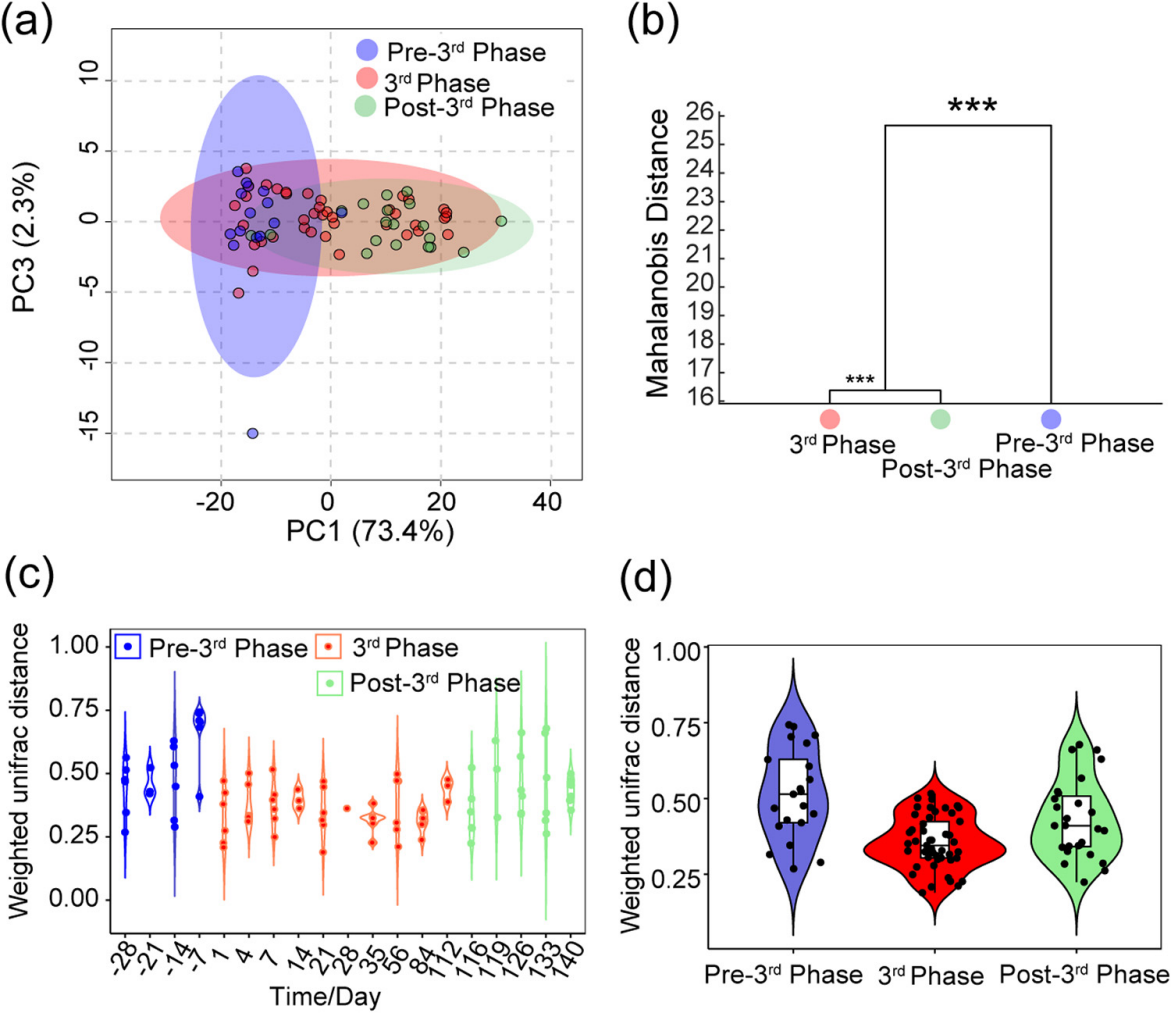
The shared living space reduced the difference in saliva microbiota among individuals. (a) PCA score plots based on the sample OTUs at the experimental phase; (b) MANOVA analysis of the different experimental phases. The statistical significance of the separation among different groups was assessed by MANOVA test based on Mahalanobis distances using the first 25 PCs of PCA at the experimental phase. The clusters were computed by applying the single linkage method to the matrix of Mahalanobis distances between the group means. (c, d) Violin maps of weighted UniFrac distances for crewmembers at the same time or at the same experimental phase. The differences between groups were compared by Kruskal-Wallis rank sum test. *, *P < *0.05; **, *P < *0.01; ***, *P < *0.001.

Variation in alpha diversity (i.e., for each crewmember) of salivary microbiota over time showed apparently random fluctuations, featuring only large variation within certain time periods (Fig. S5a) such than differences among time points and experimental phases were not significant (Kruskal-Wallis rank sum test, Fig. S5b: *KW* = 16.9120, *P*=0.5292; Fig. S5c: *KW* = 2.3652, *P*=0.3065). We visualized the overall changes of salivary microbiome over the experimental phase using PCA (Fig. 2a) and determined that the three experimental phases could not be separated. We further performed MANOVA to statistically compare differences between experimental phases. Hierarchical clustering was generated based on Mahalanobis distances as a result of MANOVA (Fig. 2b). This MANOVA analysis showed significant differences (*P*<0.001) among the three phases. Thus, to further observe the effect of shared living environments on salivary microbiota, we compared weighted UniFrac distances among crewmembers at different time points and phases. There was no significant difference at each time point (Kruskal-Wallis rank sum test, Fig. 2c: *KW* = 32.4100, *P*=0.0197), but the weighted UniFrac distance decreased in the third phase. It is worth noting that weighted UniFrac distances at each phase were significantly different, and that the third phase was significantly lower compared to the other two phases outside LP1 (Kruskal-Wallis rank sum test, Fig. 2d: *KW* = 18.1130, *P*=0.0001). This indicates that shared living spaces reduce individual differences in salivary microbiota.

**FIG 3:**
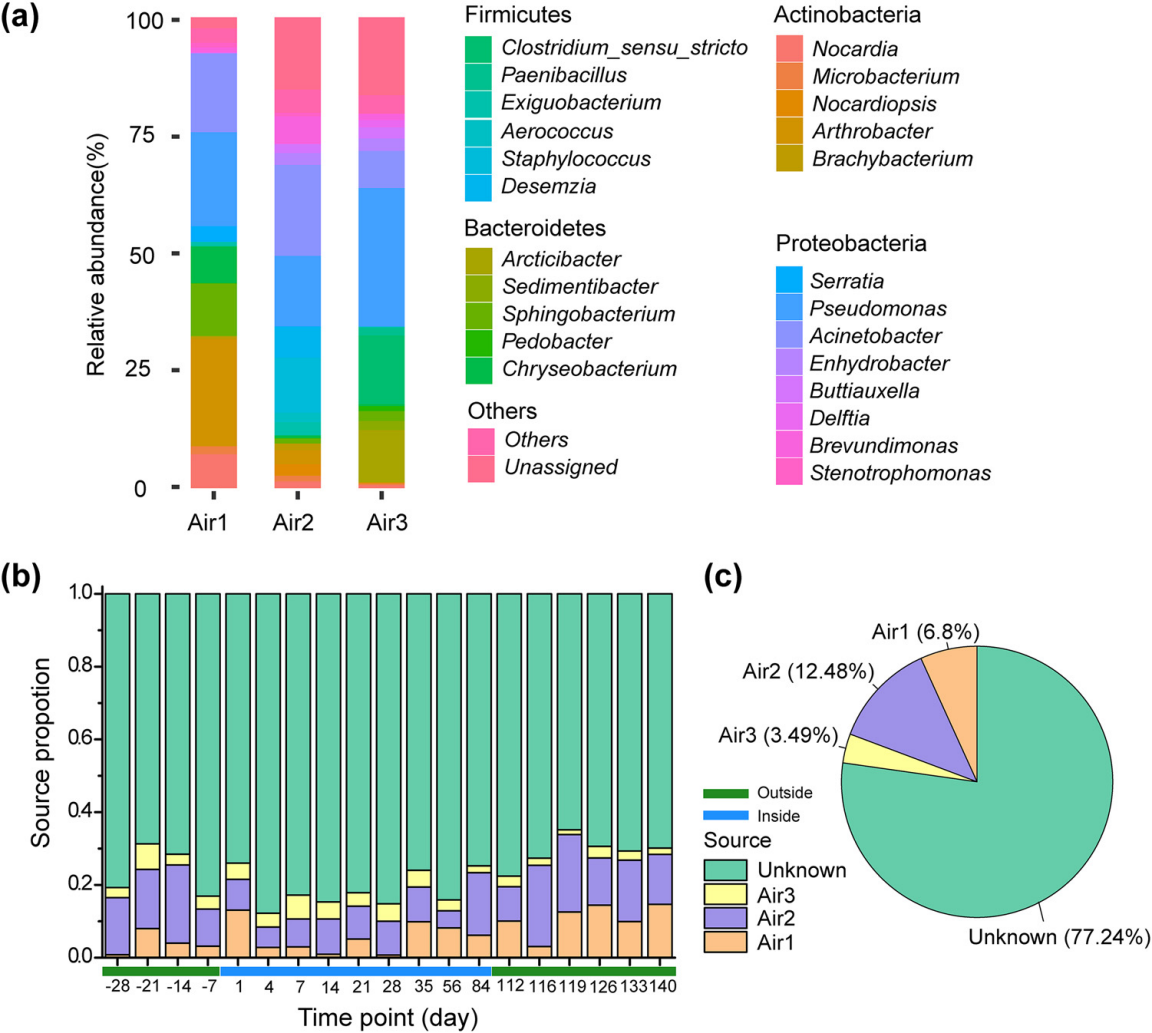
The potential impact of air microbiota on the crewmembers' salivary microbiome. (a) Changes of air microbial composition in the enclosed environment. (b) Proportion of air microbiota sources in saliva samples at different time points using the microbial source tracking FEAST. (c) Mean proportion of the air microbiota sources in all saliva samples using FEAST.

In order to analyze the role of environmental microbiota in reducing individual differences in the shared living environment, three air samples inside LP1 from the early (Air1), middle (Air2), and late (Air3) stages of the enclosed experiment were collected for 16S rRNA gene sequencing. As shown in Fig. 3a, the most abundant phyla of air microbiome in the controlled environment were Firmicutes, Bacteroidetes, Actinobacteria, and Proteobacteria, among which Proteobacteria had the highest prevalence. At the genus level, the composition of air microbiome changed greatly over time. Among them, the relative abundance of the genera *Nocardia*, *Arthrobacter*, *Sphingobacterium*, and *Chryseobacterium* decreased over time, and that of *Arcticbacter* and Staphylococcus increased over time (Fig. 3a). To determine the overlapped OTUs between air and saliva samples, the microbial source tracking method FEAST (30) was used. We considered three air samples inside LP1 as sources and the crewmembers’ saliva samples at different time points as sinks. As shown in Fig. 3b, at the first time point upon entering LP1, the salivary microbiome of crewmembers was mainly derived from the air at the early stage of the enclosed experiment (Air1), while at other time points, the salivary microbiome of crewmembers was mainly derived from the air of the middle stage (Air2). In summary, the FEAST analysis revealed that the proportion of bacterial community from the air source was about 23% for the saliva, indicating the air microbiota may play a role in reducing individual differences in the shared living environment (Fig. 3c). Inside the LP1, 30 specific genera were found in common between in the air and saliva samples from the VENN diagram (Fig. S6).

### Changes in salivary microbiota in the enclosed environment.

**FIG 4:**
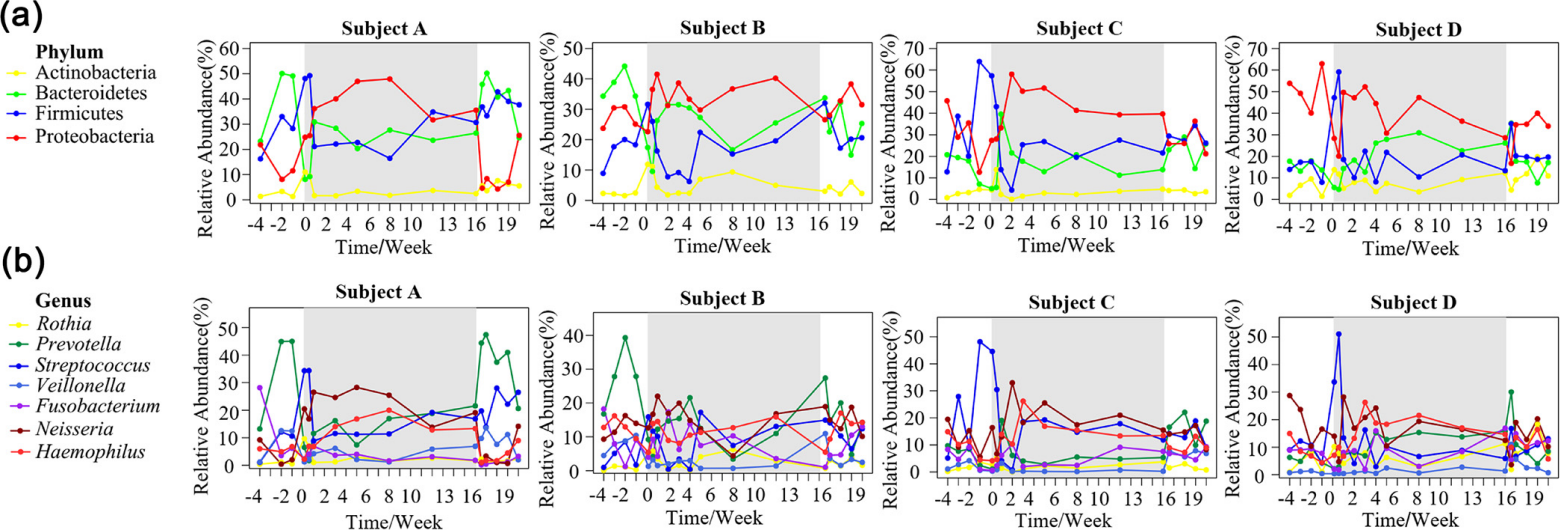
Dynamics of crucial phyla (a) and genera (b) in crewmembers’ saliva throughout the experiment. Each curve represents a phylum or genus, and shadows indicate that the crewmembers are in LP1.

We selected the relative abundance of the core phyla and genera to plot the curves over time, as shown in Fig. 4. The results indicated that the enclosed environment was related to the changes in the relative abundance of salivary microbiota. The representation of different phyla and genera showed greater variability before and after time in the BLSS than during time in the BLSS. At the phylum level, Actinobacteria showed a transient increase during the first week after crewmembers entered LP1 (Fig. 4a). In the LP1, the abundance of Actinobacteria in subjects B and D also fluctuated. In the same period, Bacteroidetes decreased significantly before entering LP1, and then increased rapidly. After the crewmembers left LP1, the abundance of Bacteroidetes basically returned to the previous level (except for subject B, Fig. 4a). Regarding Proteobacteria, its relative abundance in subject A gradually increased after entering LP1, and then gradually decreased after leaving LP1 and returned to the level before entering LP1; subject B and D maintains a fairly constant level across the entire experiment, while subject C showed an increase followed by a gradual drop while in the LP1 (Fig. 4a). For Firmicutes, its relative abundance showed an increase followed by a rapid drop after entering LP1. Then, the relative abundance of Firmicutes in subjects A and B gradually increased, and subjects C and D maintained a fairly constant level (Fig. 4a). At the genus level, *Rothia* was the most abundant of Actinobacteria (59.2 ± 23.4%), and its trend was consistent with Actinobacteria (Fig. 4b). *Prevotella* was the most abundant of Bacteroidetes (69.4 ± 18.3%), and its change trend was consistent with that of Bacteroidetes. Streptococcus and *Veillonella* were the most abundant of Firmicutes (51.4 ± 18.7%, 15.6 ± 14.2%), and the change trend of Streptococcus was in accordance with Firmicutes. Meanwhile, the abundance of *Veillonella* decreased slightly after crewmembers entered LP1 and recovered after they left LP1 (Fig. 4b). *Neisseria* and Haemophilus were the most abundant of *Proteobacteria* (43.8 ± 14.6%, 35.2 ± 12.8%). The abundance of *Neisseria* first increased and then decreased in crewmembers’ saliva, including subject D, after entering LP1, reaching the highest level of about 1.73∼7.28 times of that outside of LP1 within 5 weeks (Fig. 4b). Meanwhile, the abundance of Haemophilus changed consistently with *Neisseria*, except for subject B (Fig. 4b).

### Changes of salivary cytokines and their correlation with salivary microbiota.

We adopted ACF to analyze the temporal dynamics of salivary cytokines (IL-1β, IL-6, IL-10, TNF-α, and IFN-γ) throughout the experiment (26, 27). The autocorrelation analyses showed that none of the cytokines showed significant autocorrelation (Fig. S7), and they were stationary stochastic processes, suggesting that the crewmembers’ salivary cytokines exhibited no reliable changes with time during the whole experiment. Furthermore, using the Mann-Kendall trend test (28, 29) to analyze whether there was a trend change for the continuous period from normal environment into LP1, as shown in Table S3, IL-1β showed an increasing trend (except for subject A), IL-6 (except for subject B), IL-10, and TNF-α showed a decreasing trend, and IFN-γ kept a consistent level. In specific, TNF-α showed a tendency to decrease significantly (*P*<0.05), except for subject C.

**FIG 5:**
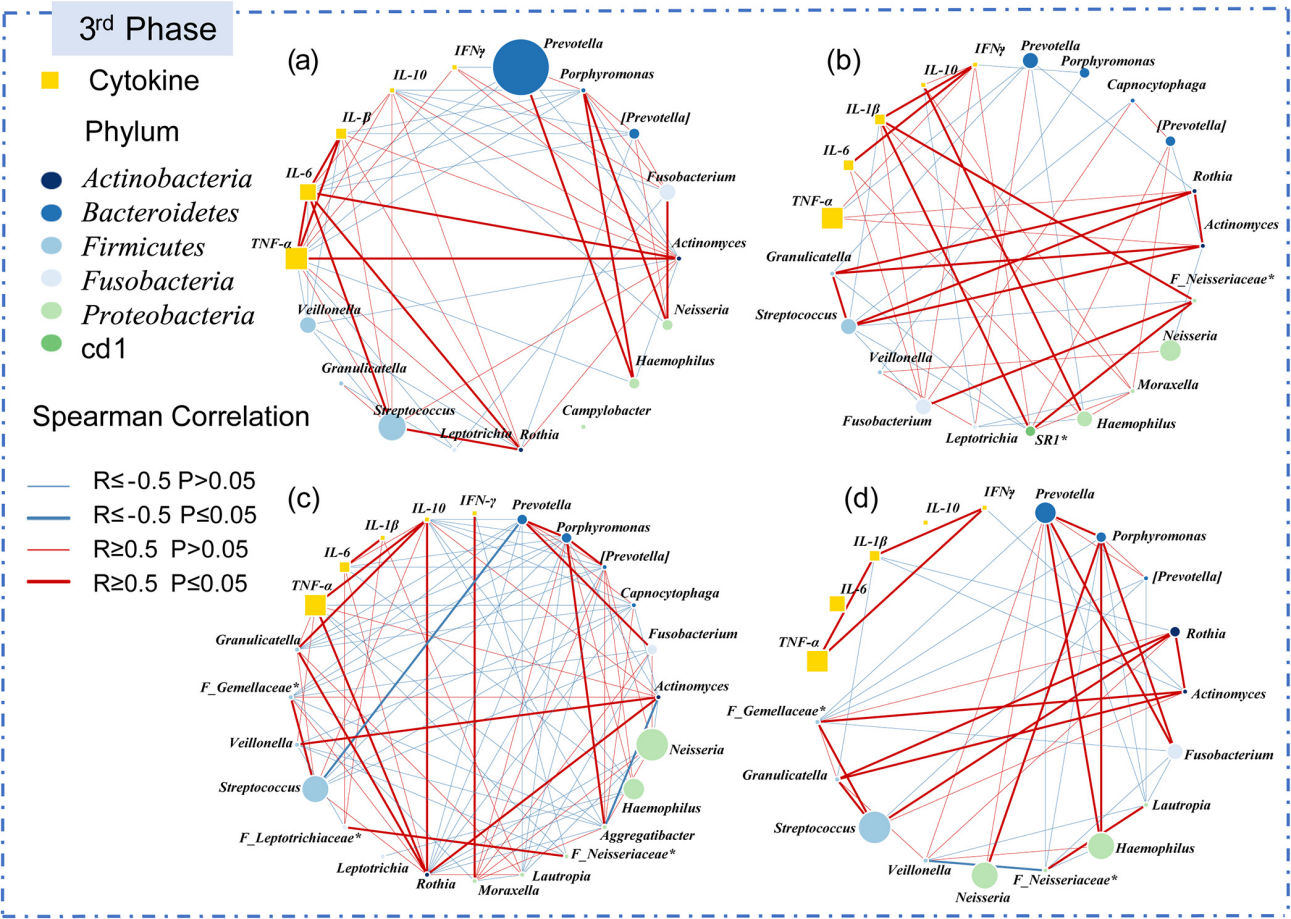
Co-occurrence network of crewmembers’ salivary cytokines and highly abundant microbial genera in LP1. The co-occurrence networks of the subject A, B, C, and D were shown in figures (a), (b), (c), and (d), respectively. Select a genus with relative abundance over 1%, calculate the Spearman’s correlation coefficient of saliva microbiota and cytokines in LP1, and then generate the graph using R (ver 3.5.3) packages (igraph). Each node represents a microbial genus or a salivary cytokine. The circles colored by phylum represent different genera, and the squares represent different cytokines. The size of the nodes is proportional to the microbial abundance or salivary cytokine level. The link between nodes indicates Spearman’s correlation (|r| ≥ 0.5). The blue and red lines indicate negative correlation and positive correlation, respectively. Specifically, the bold font indicates significant correlation (*P ≤ *0.05).

We analyzed the Spearman correlation between the highly abundant salivary microbial genera and the salivary cytokines of each crewmember in LP1 (Table S4), as shown in Fig. 5. The co-occurrence network of four crewmembers have strong individual characteristics, which is the most complex for subject C in terms of the correlation among saliva microbiota and cytokines. Notably, TNF-α shows a consistent positive correlation with *Actinomyces* and *Rothia* (*R* ≥ 0.5), which is observed in subjects A, B, and C, but not D.

## DISCUSSION

During long-term space missions, health problems such as symbiotic microbial disorders and impaired immunity often occur because of psychological and environmental pressures, which may in turn affect the success of such missions. The “Lunar Palace 365” project was a multicrew and enclosed experiment carried out in LP1, representing a unique and invaluable opportunity to study symbiotic microbial disorders and immune adaptation problems that humans may face in long-term space missions. In this context, we explored the temporal dynamics of salivary microbiota and salivary cytokines in the four crewmembers participating in the third phase of “Lunar Palace 365,” including the period before entering LP1 (28 days), for about 200 days during the experiment, and after their return to regular life (30 days). Previous studies had already observed changes in oral microbiota (16), infectious diseases (17), and compromised or impaired immunity (5) during space missions. Researchers believed that these were mainly caused by cosmic radiation, microgravity, and the long-term consumption of pre-packaged food. In the “Lunar Palace 365” project, we kept the crewmembers’ diet consistent with that in regular life by planting food crops, and studied the effects of enclosed controlled environment on the human salivary microbiota and oral immunity for the first time.

Salivary microbiota is more stable than other symbiotic microbiota (31) and shows no significant changes within 1 year in adults (32). Our results are consistent with previous studies, and the dominant species steadily fluctuated during the experiment, with no significant tendency. Besides, there was no significant difference in the alpha diversity of the four crewmembers’ salivary microbiota between sampling times and phases, indicating that the crewmembers were well-adapted to the enclosed environment, and their salivary microbial community remained stable. Though the oral microbiome of a single person is relatively stable, it can be significantly different among individuals (33). Nasidze et al. (34) analyzed the saliva samples of 120 healthy people from 12 countries and regions worldwide and found that the oral microbiota was highly person-specific. In Mars500 mission, Bacci et al. (15) found that the salivary microbiota of the subjects also showed individualized characteristics and stability.

In this study, we also found that the environment within BLSS tends to stabilize the oral microbiome relative to the environment outside BLSS. These results may be caused by simple human relations prior to and after time in BLSS. For example, kissing partners outside BLSS may have a significant impact on the variability of salivary microbiome (35). In BLSS, kissing behavior should not occur because these crewmembers are not lovers. In addition, the diet in BLSS is more stable than that outside BLSS, which is one of the reasons why salivary microbiome in BLSS are more stable. However, some microbiota also showed transient trends in the enclosed environment, including the reduction of Bacteroidetes and the increase of Proteobacteria (Fig. 4a). A study based on 16S pyrosequencing found that the abundance of oral Bacteroidetes was significantly elevated in patients with periodontitis, while that of Proteobacteria was reduced (36). In addition, Said et al. (37) found that patients with inflammatory bowel disease had more abundant salivary Bacteroidetes, accompanied by reduced Proteobacteria. Based on previous research, we believe that trends of highly abundant phyla seem to be beneficial to oral and general health in enclosed environments. Moreover, we found that phylum-level changes were mainly caused by highly abundant genera. For example, the changes of *Rothia*, *Prevotella*, and Streptococcus were consistent with those of Actinobacteria, Bacteroidetes, and Firmicutes, respectively. In addition, the trends of *Neisseria* and Haemophilus were roughly the same as that of Proteobacteria. *Prevotella* exists extensively in the human microbiota, and is significantly increased in the saliva of patients with caries (38), esophagitis, sinusitis (39), inflammatory bowel disease (37), HIV with hyperviralemia (40), and bacterial vaginosis (41). In our results, *Prevotella* was reduced in crewmembers’ saliva with the exception of subject D, indicating that the salivary microbiota of crewmembers may be healthier in the studied enclosed environment. In addition, we found that *Neisseria* and Haemophilus were elevated in the enclosed environment, which was associated with type 2 diabetes (42) and oral leukoplakia (43), respectively. We suspect that this phenomenon may be related to simpler food processing steps in the enclosed environment. Several studies discovered that the oral microbiota of hunters, traditional farmers, Westerners, and vegetarians shows great variation, and finely processed foods in the diet results in less abundant *Neisseria* and Haemophilus (44, 45). Dietary changes in long-term missions play an important role in shaping the salivary microbiota. The increased abundance of Streptococcus in astronauts’ saliva during Skylab missions was thought to be caused by the higher intake of prepared foods (16). Our study did not show similar results, indicating that ensuring the supply of fresh food by BLSS helps reduce any adverse changes in the oral microbiota.

According to the weighted UniFrac beta diversity results, the bacterial communities of the four crewmembers became, to some extent, more similar to each other over time, indicating that the shared living space reduces the individual differences in the salivary microbiota. Our previous 105-day closed experiment (24) and the Mars500 ground-based space simulation (46) showed similar phenomena for human intestinal microbes. Our result is in agreement with previous studies in that, small-scale effects due to shared living spaces can significantly affect its composition (47). Stahringer et al. (48) observed the same effect in the salivary microbiome, and also found that the salivary microbiome of twins became less similar as they grew older and ceased cohabiting, concluding that “nurture trumps nature” in the salivary microbiome. In this study, the FEAST analysis results indicated that, in the shared living space, air microbiome may play an important role in reducing the individual differences in salivary microbiota. Our work supports the interaction between environmental microbiome and salivary microbiome (Fig. S6), and suggests that another important factor in long-term persistence may be the regular reseeding of the ecosystem with bacteria from the external environment. This also reminds us of the importance of crewmembers’ oral microbiota and environmental microbes in long-term space missions, as well as the necessity to prevent the breeding of harmful microbiota in space.

Cytokines are important components in saliva, participating in host defense to maintain oral and systemic health, and providing information about local and systemic conditions (49, 50). Our study found that salivary cytokines fluctuated smoothly throughout the experiment. However, after the crewmembers entered the enclosed environment from a standard environment, IL-1β showed an increasing trend, IL-6 and IL-10 presented a decreasing trend, and TNF-α had a significant downward trend. Changes in the salivary cytokines may be caused by oral microbes. For instance, increased periodontal pathogen abundance will enhance the stimulation of TLRs, induce Th1 to secrete IL-2, IFN-γ, TNF-α to kill intracellular infection pathogens, and then induce Th2 to secrete IL-4, IL-5, IL-6, IL-13 to regulate humoral immunity and limit Th1 response (51). On the other hand, oral symbiotic microbiota can trigger the secretion of IL-10 through T-reg cells and inhibit the activity of effector T cells (52). The crewmembers’ salivary cytokines showed a decreasing trend in the studied enclosed environment, indicating that there was no pathogenic changes in saliva microbiota and subsequent abnormal inflammatory response. This may be related to the large number of grown plants in LP1. Long-term plant care can help crewmembers generate positive emotions (23), and a high-plant and high-fiber diet may also be beneficial to maintain crewmembers’ health (24). In addition, IL-1β is a pro-inflammatory cytokine stimulated by bacterial lipopolysaccharides, which participates in the inflammatory response and stimulates immune cells to secrete IL-1β, IL-2, IL-6, IL-8, TNF-α, and IFN-γ (53, 54). Schbacher et al. (55) reported that the response of IL-1β to stress has predictive value for mental health. Szabo et al. (56) also found that psychosocial stress was associated with higher salivary IL-1β and increased negative emotions caused an increased IL-1β (57). Furthermore, maintaining positive emotions can alleviate the negative effects of stress by reducing the inflammatory response (58). Therefore, it was assumed that the increase in IL-1β in this study might have been caused by psychosocial stress and increased negative emotions (23), and was not a manifestation of the increased oral inflammation of the crewmembers.

Previous studies revealed strong correlations between certain inflammatory biomarkers and salivary microbiota compositions, for instance, that IL-17 plays an important role in oral antifungal activity (59). Besides, Said et al. (37) found that lower lysozyme and elevated IL-1β, IL-8, and IgA in the saliva of patients with inflammatory bowel disease were likely to be synergistically or interactively associated with the abundance of Streptococcus, *Prevotella*, *Veillonella*, and Haemophilus. Porphyromonas gingivalis can colonize atherosclerotic plaques, stimulating the secretion of inflammatory factors such as TNF-α, IL-1β, and IL-6, and causing inflammation and vascular endothelial damage (60). We found complex correlations among salivary cytokines and highly abundant genera in the enclosed environment, and TNF-α showed a consistent correlation with *Actinomyces* and *Rothia*. *Actinomyces* is an early colonizer in the process of plaque maturation. Sato et al. (61) found that peptidoglycans of Actinomyces naeslundii can cause the secretion of IL-1β, IL-6, and TNF-α. The positive correlation between TNF-α and *Actinomyces* in our study confirms their research, and also suggests that decreased TNF-α in the saliva of crewmembers in an enclosed environment may be related to decreased *Actinomyces*. Furthermore, *Rothia* is a conditional pathogen widely occurring in the human oral cavity, with the most typical species of Rothia dentocariosa. Kataoka et al. (62) found that *R. dentocariosa* can induce TNF-α production through TLR2, which is consistent with our findings. Our results suggest that there is also a certain level of correlation between salivary microbiota and salivary cytokines in healthy individuals, which can be disturbed by the environment encountered in daily life. Therefore, when studying the correlation between salivary microbiota and salivary cytokines, we should consider the host’s health status, diet and living environment to obtain more accurate results.

In conclusion, our study proves that environmental isolation will not cause salivary microbiota and immune disorders, and salivary microbiota and cytokines in an enclosed environment showed no regular change pattern in the long-term. Throughout the experiment, although salivary microbiota and cytokines transiently fluctuated after crewmembers entered the enclosed environment, the levels for most of them returned to previous values after returning to the previous environment, indicating that crewmembers adapted well to the environmental isolation without permanent changes. The shared living space reduced the individual differences in the salivary microbiota, which may be due to the significant influencing role of the air microbiota. Although salivary IL-1β has a tendency to increase in an enclosed environment, this trend is more likely to be a response to prolonged stress. Overall, the crewmembers’ salivary inflammatory cytokines were reduced in the enclosed environment. The correlation between salivary cytokines and microbiota in environmental isolation indicates that the oral mucosal barrier of healthy individuals may be sensitive to changes in their salivary microbiota. Our study presents a new aspect of the impact of isolation cabins on crewmembers’ health to assist with future research, and provides guidance for studying the effect of enclosed controlled environments on human immunity based on salivary microbiota as an indicator.

## MATERIALS AND METHODS

### Participants.

The participants in this study comprised four crewmembers involved in the third phase of the Chinese “Lunar Palace 365” project, including two males (subject A, 25 years old; subject B, 30 years old) and two females (subject C, 25 years old; subject D, 29 years old). No crewmembers had any history of smoking, oral disease, serious illness, or chronic disease. The bulk of general physical examination indices (performed at the 306th Hospital of People’s Liberation Army, Beijing, China) before and after the third phase were within the normal ranges. All crewmembers had healthy oral cavities.

### Study design.

**FIG 6:**
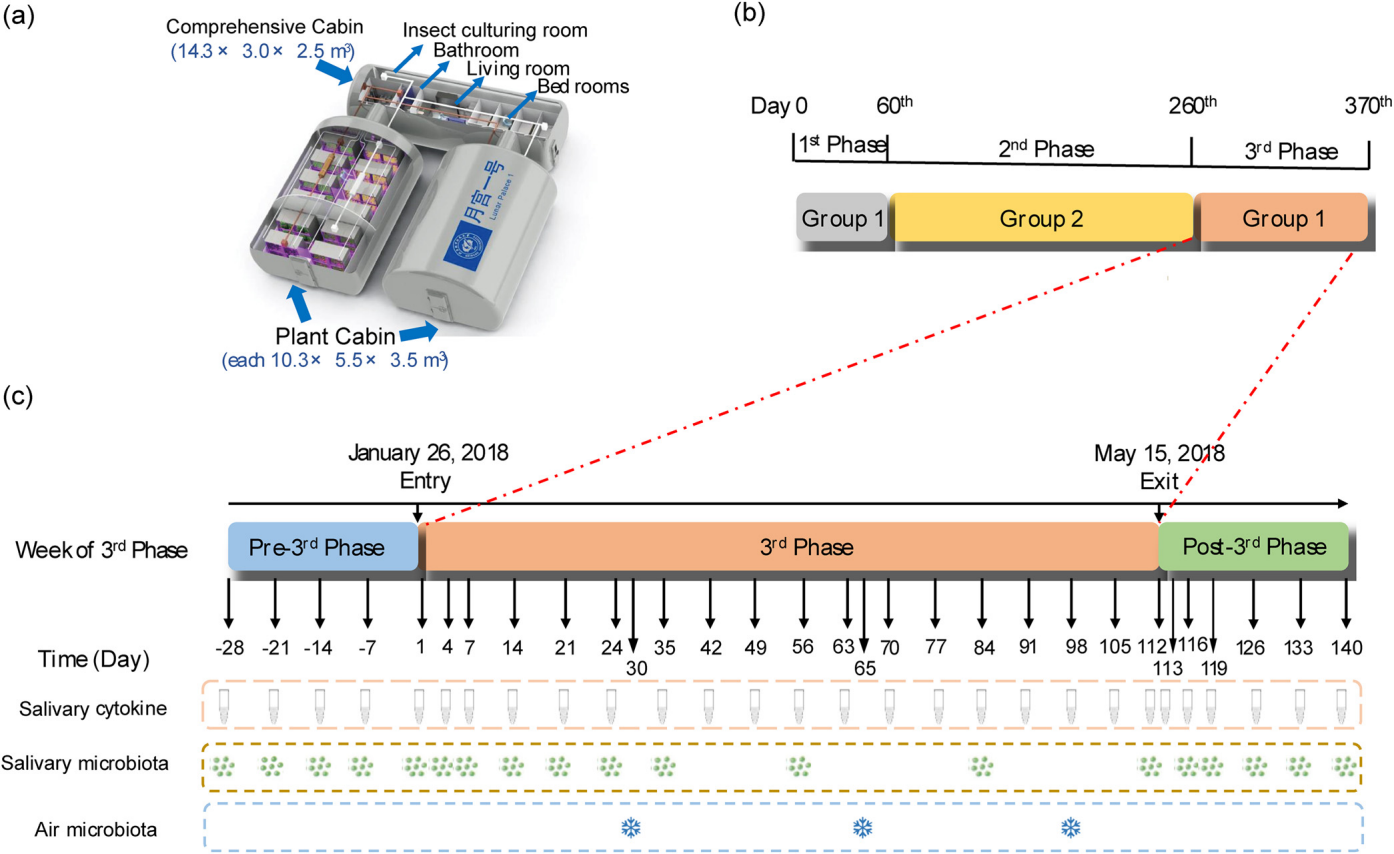
Experimental design and procedures. (a) Structure of “Lunar Palace 1”; (b) The mission of “Lunar Palace 365” project; (c) Experimental design; tube: sampling of crewmembers’ salivary cytokines; eight green dots: sampling of crewmembers’ salivary microbiota; blue snowflakes: sampling of air microbiota.

The “Lunar Palace 365” project was a 370-day multicrew enclosed experiment carried out in a ground-based experimental BLSS platform named LP1, located at the Institute of Environmental Biology and Life Support Technology, Beihang University, Beijing, China. LP1 is a highly enclosed ecosystem integrating the efficient cultivation of higher plants, animal protein production, urine nitrogen recycling, and bioconversion of solid waste (21). LP1 consists of a comprehensive cabin and two plant cabins with a total area of 160 m^2^ and a total volume of 500 m^3^. The comprehensive cabin has four private bedrooms, a living room, a bathroom, and an insect culturing room (Fig. 6a). The “Lunar Palace 365” project was divided into three phases completed by two groups (group 1 and group 2) The first phase lasted for 60 days with four crewmembers from group 1, while the second phase lasted for 200 days with four crewmembers from group 2, and the third phase lasted for 110 days with four crewmembers from group 1 (Fig. 6b). We explored the temporal dynamics of the salivary microbiota and cytokines in group 1 participating in the third phase of the “Lunar Palace 365” project, including 1 month before entering LP1 and 1 month after returning to regular life. The experimental design is shown in Fig. 6c.

### Collection of whole saliva and determination of cytokines.

All saliva samples were collected between 9:30 p.m. and 10:30 p.m. to minimize circadian variation in salivary composition. One hour before sampling, crewmembers were not allowed to eat or drink (except water) and perform oral hygiene activities, including tooth brushing. Saliva was collected using the spitting method for 10 min. Cellular debris in saliva samples were removed by centrifugation at 4,000 × *g* for 20 min at 4°C, and supernatants were aliquoted and stored at −80°C until further analyses. Cytokines (IL-1β, IL-6, IL-10, TNF-α, IFN-γ) in saliva were quantified using Enzyme-linked Immunosorbent Assay (Beijing RGB Technology Development Co., Ltd., Beijing, China).

### Collection of saliva and air samples for Illumina HiSeq sequencing.

Saliva samples and air samples were collected at the time set in Fig. 6c. The saliva samples for Illumina HiSeq sequencing were collected immediately after getting up in the morning according to the instructions: (i) relax cheeks and gently rub for 30 s to produce saliva; (ii) spit saliva into a 5-mL cryotube and collect about 1 mL of saliva; (iii) add 2 mL of phosphate-buffered saline (PBS) to the cryotube and mix it by inversion 10 to 20 times; (iv) store the cryotube in a −20°C freezer for further testing. No consumption of food or drink other than water was allowed within 2 h before saliva sampling, and oral hygiene activities were also forbidden.

Three air samples from the early, middle, and late stages of the third phase of the “Lunar Palace 365” experiment were collected for Illumina HiSeq sequencing. Air samples were taken using the filter membrane of the air purifier in the comprehensive cabin of LP1. Subsequent to the use of the aseptic technique and operating conditions, the filter membrane was removed from the air purifier, washed with PBS, and samples were then stored in a −20°C refrigerator for subsequent genetic sequencing analysis.

Total bacterial DNA was extracted from the samples using the Power Soil DNA isolation kit (MO BIO Laboratories) according to the manufacturer’s protocol. The V3 to V4 region of the bacterial 16S rRNA gene was amplified using 341F (5′-CCTAYGGGRBGCASCAG -3′) and 806R (5′-GGACTACNNGGGTATCTAAT -3′) primers (63). The PCR amplification was performed in a total volume of 50 μL, which contained 10 μL 10 × PCR buffer, 0.2 μL Q5 High-Fidelity DNA polymerase, 10 μL High GC Enhancer, 1 μL dNTP, 10 μM each primer, 50 ng genome DNA, and ddH_2_O to supplement the remaining volume. Thermal cycling conditions were as follows: pre-denaturation at 95°C for 5 min, followed by 15 cycles of denaturation at 95°C for 1 min, renaturation at 50°C for 1 min, and extension at 72°C for 1 min, with a final extension at 72°C for 7 min. PCR products from the first step were purified through VAHTS DNA Clean Beads (Vazyme, cat. N411, Nanjing, China). A second round of PCR was performed in a 40 μL reaction volume containing 20 μL 2×Phμsion HF MM (Thermo scientific, USA), 8 μL ddH_2_O, 10 μL of each primer, and 10 μL PCR products from the first step. Thermal cycling conditions were: pre-denaturation at 98°C for 30 s, followed by 10 cycles of denaturation at 98°C for 10 s, renaturation at 65°C for 30 s, extension at 72°C for 30 s, and final extension at 72°C for 5 min. All PCR products were quantified using Quant-iT dsDNA HS Reagent and pooled together at a quality ratio of 1:1. Finally, 1.8% agarose gel electrophoresis was performed. The high-throughput sequencing analysis was conducted on the purified, pooled sample using the Illumina HiSeq 2500 platform (2 × 250 paired ends) at Biomarker Technologies Corporation, Beijing, China.

The raw sequence was spliced and filtered using FLASH (version 1.2.11) (64) and Trimmomatic (version 0.33) (65) successively, and the chimera was removed using UCHIME (version 8.1) (66) to obtain high-quality Tags sequences. Quality-checked 16S rRNA gene sequences were classified into OTUs within 0.03 difference (equivalent to 97% similarity) using USEARCH (version10.0) (67). The taxonomic classification at different taxonomic levels of these OTU sequences was carried out using RDP classifier (version 2.2, http://sourceforge.net/projects/rdpclassifier/) (68) against the Silva (Release 128, http://www.arb-silva.de) (69) and UNITE (Release 7.2, http://unite.ut.ee/index.php) (70) with a 80% confidence threshold (71). A phylogenetic tree was constructed at the genus level based on PyNAST (version 1.2.2, http://biocore.github.io/pynast/) (72) and ClustalW2 (http://www.ebi.ac.uk/Tools/msa/clustalw2/) (73), and a distance matrix was calculated among samples.

Sequencing-based 16S rRNA surveys are usually normalized by converting OTU sequence counts into fractional abundances for each sample. However, this standard technique leads to compositional effects (74), which may cause false relationships between OTUs, or between OTUs and cytokines. The analysis of saliva microbiota dynamics over the whole experiment adapted the normalization technique developed by David et al. (25). Briefly, for each crewmember, (i) time points were normalized in the standard manner so that the sum of all fractional OTU abundances at a given time point was 1; (ii) highly abundant OTUs were selected accounting for 90% of median time point reads; (iii) each time point was normalized to a reference community that was computed for each sample based on other time points with a similar community structure. Reference OTU values were computed using a weighted median across time series, with time point weights set to be (1 - *j*)^2^ and with *j* being the pairwise Jensen-Shannon Distance (JSD) score of the sample being normalized (25).

### Statistical analyses.

All statistical analyses were conducted using R and MATLAB. However, as crewmember salivary microbiota and salivary cytokines as variables are susceptible to other biotic and abiotic units, a set of hypothetical stochastic differential equations could be used to express the influencing mechanisms of biotic and abiotic factors on the dynamic response of these variables. If a well-designed BLSS could provide sufficient sustenance support for crewmembers, they could acclimate themselves to the enclosed environment. Thus, under this assumption, changes in crewmembers’ salivary microbiota and cytokines should be a stationary stochastic process. We adopted the ACF (26, 27) to develop this assumption by evaluating whether ACF would be only dependent on the time interval rather than time. If the variation in the two factors was a stationary stochastic process, we used the Mann-Kendall trend test (28, 29) to analyze whether there is a trend change between the normal environment and LP1. As for the differences among groups in their salivary microbiota, PCA and multivariate analysis of variance (MANOVA) were performed using R packages (reshape 2, ggplot 2 and vegan) and MATLAB (the MathWorks Inc.), respectively. Fast expectation-maximization microbial source tracking (FEAST) was employed to estimate the proportion of air sources contributing to the salivary microbiota of crewmembers (30). Furthermore, we analyzed the alpha and beta diversity of salivary microbiota at different time points during the experimental phases. Depending on the normality and variance homogeneity of the data, one-way ANOVA or Kruskal-Wallis rank sum test was used, and the *P* values were corrected for multiple testing using the Bonferroni method (75). The relationships among salivary microbiota and cytokines were analyzed by Spearman’s correlation tests using R (ver 3.5.3) packages (hmisc), and adjusted for multiple comparisons by the FDR correction method.

### Ethical statement.

We obtained written informed consent from four subjects enrolled in the study. This study was approved by the Science and Ethics Committee of School of Biological Science and Medical Engineering in Beihang University, Beijing, China (Approval ID: BM20180003) and complied with the Helsinki Declaration.

### Data availability.

The Illumina HiSeq raw sequence data reported in this paper have been deposited in the National Centre for Biotechnology Information Sequence Read Archive (NCBI SRA; BioProject ID: PRJNA611700). Other published datasets used in this work have been listed in Supplementary information.
