# Supplementary material for: Effects of Long-Term Enclosed Environment on Human Health Based on the Analysis of Salivary Microbiota and Cytokines
Source: Microbiol Spectr. 2022 Mar 7;10(2):e00254-22. doi: 10.1128/spectrum.00254-22 (PMC9045383; doi:10.1128/spectrum.00254-22)
Supplement: SUPPLEMENTAL FILE 1 — Supplemental material. Download SPECTRUM00254-22_Supp_1_seq9.pdf, PDF file, 1.2 MB [file spectrum00254-22_supp_1_seq9.pdf]

Supporting information

Effects of long-term enclosed environment on human health based on the analysis of salivary microbiota and cytokines

Zikai Hao [a,d,1], Yinzhen Zhu [a,b,1],Yuming Fu, [a,b,c], Jianlou Yang [a,b], Chen Meng [a,b], Chen Dong [e*], Hong Liu [a,b,c,*]

a Beijing Advanced Innovation Centre for Biomedical Engineering, Beihang University, Beijing 102402, China

b Institute of Environmental Biology and Life Support Technology, School of Biological Science and Medical Engineering, Beihang University, Beijing 100083, China

c International Joint Research Center of Aerospace Biotechnology & Medical Engineering, Beihang University, Beijing 100083, China

d State Key Laboratory of Software Development Environment, School of Computer Science and Engineering, Beihang University, Beijing, China

e Laboratory of Sport Nutrition and Intelligent Cooking, Shandong Sport University, Jinan 250102, China

1 Zikai Hao and Yinzhen Zhu contributed equally to this work and should be considered co-first authors, Author order was determined by alphabetical order of their last names.

* Both authors are correspondence authors

Chen Dong, Laboratory of Sport Nutrition and Intelligent Cooking, Shandong Sport University, Jinan 250102, China. Email: dongchen@sdpei.edu.cn

Hong Liu, Lab of Environmental Biology and Life Support Technology, School of Biological Science and Medical Engineering, Beihang University, Beijing, 100191, China. Email: lh64@buaa.edu.cn

Supplementary Fig. 1

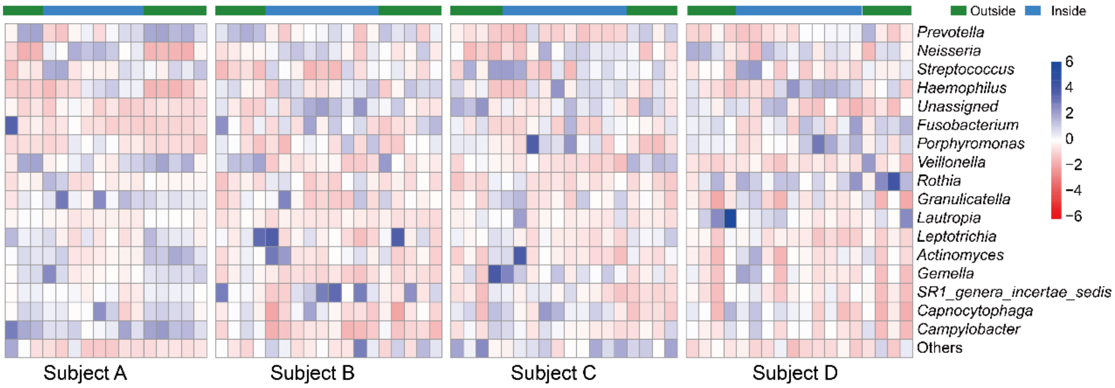

Supplementary Fig S1: Heatmap of the relative abundance variation of highly abundant genera over time in the salivary microbiota. The genera with average relative abundance greater than 1% in each sample were selected as highly abundant genera.

Supplementary Fig. 2

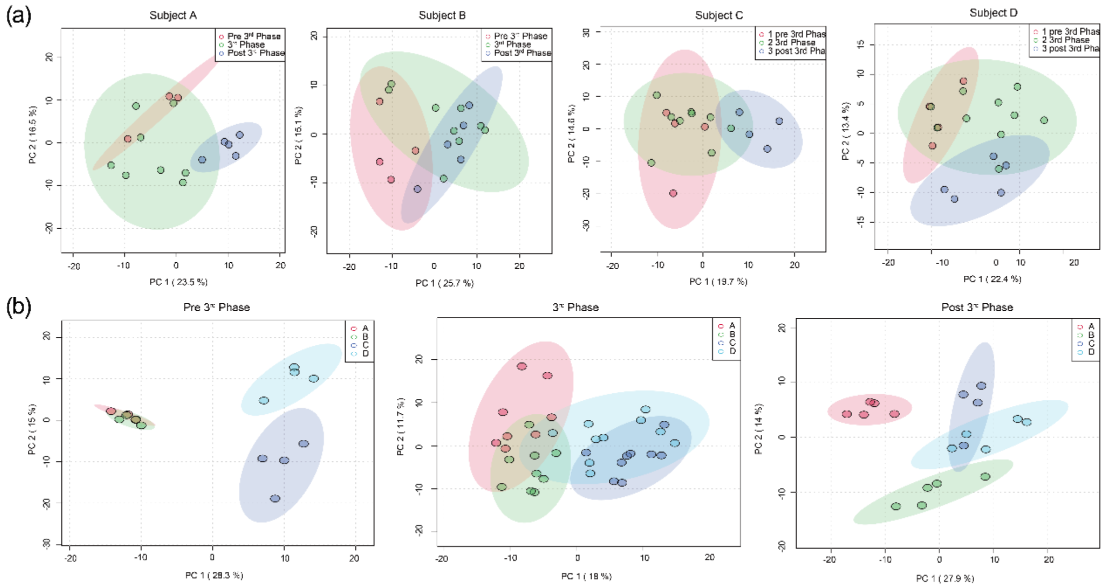

Fig S2: The principal component analysis (PCA) score plots based on OTUs of the salivary microbiota. (a) The PCA score plots based on OTUs of the different individual samples at the different experimental phase. (b) The PCA score plots based on OTUs of the different experimental phase samples with the different individual.

Supplementary Fig. 3

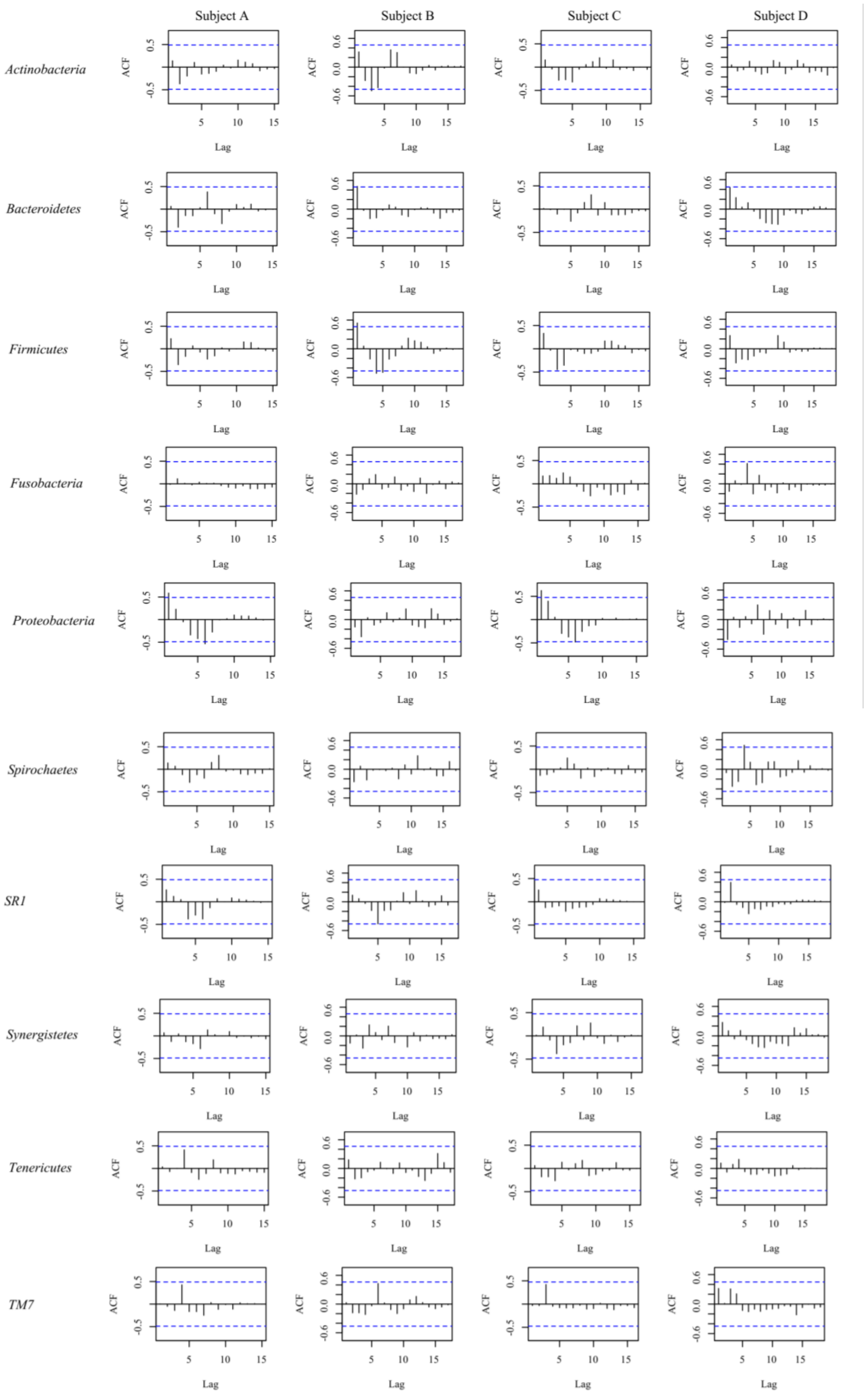

Fig S3: The sample autocorrelation function plots (ACF) of the highly abundant phyla in crewmembers' saliva. Blue lines indicate upper and lower range of confidential region, sample autocorrelation that falls into the confidential region indicate stability of the phylum. The results showed there was no consistent significant autocorrelation for crewmembers and the crewmembers' saliva microbiota variations at phylum were stationary stochastic process.

Supplementary Fig. 4

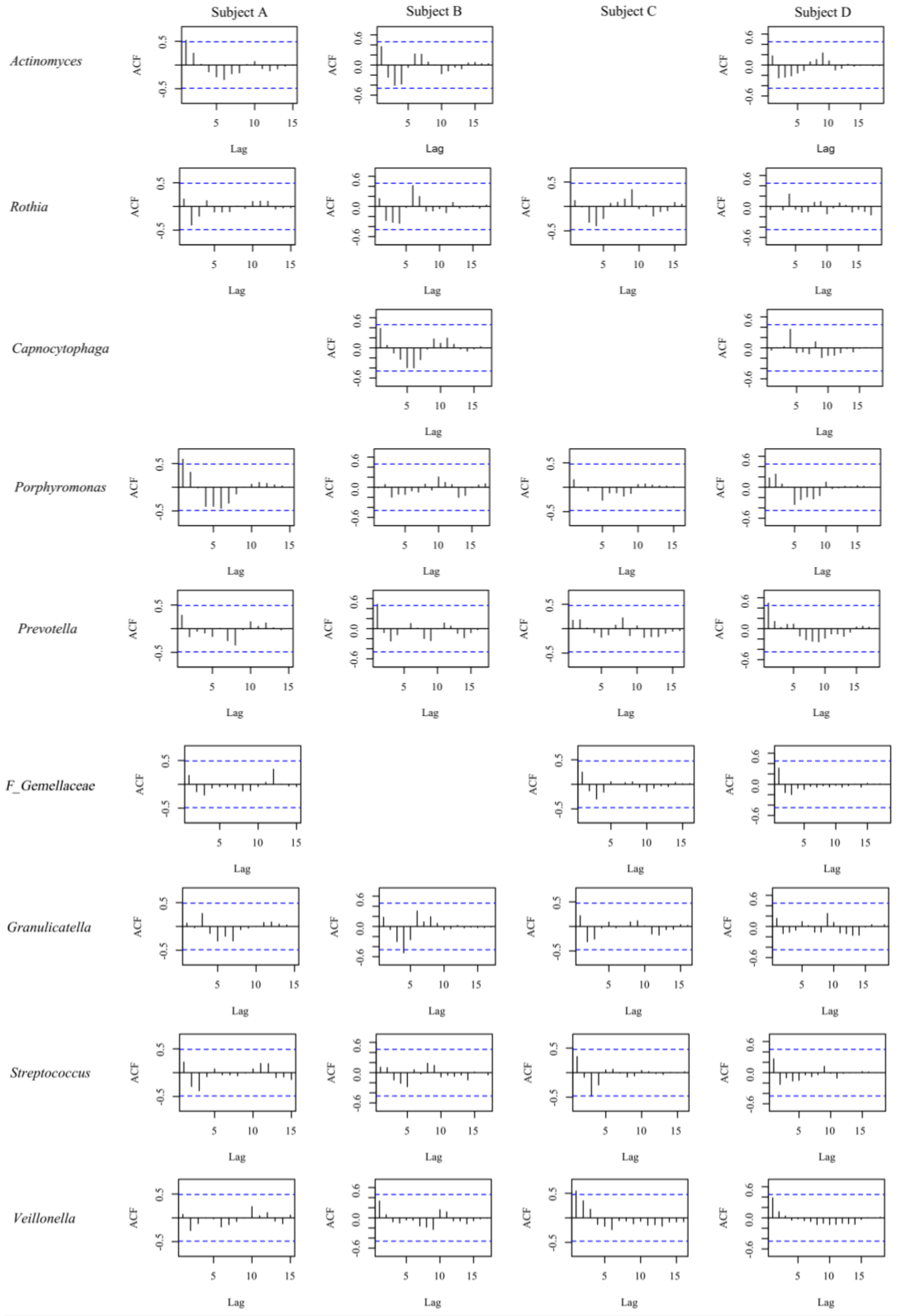

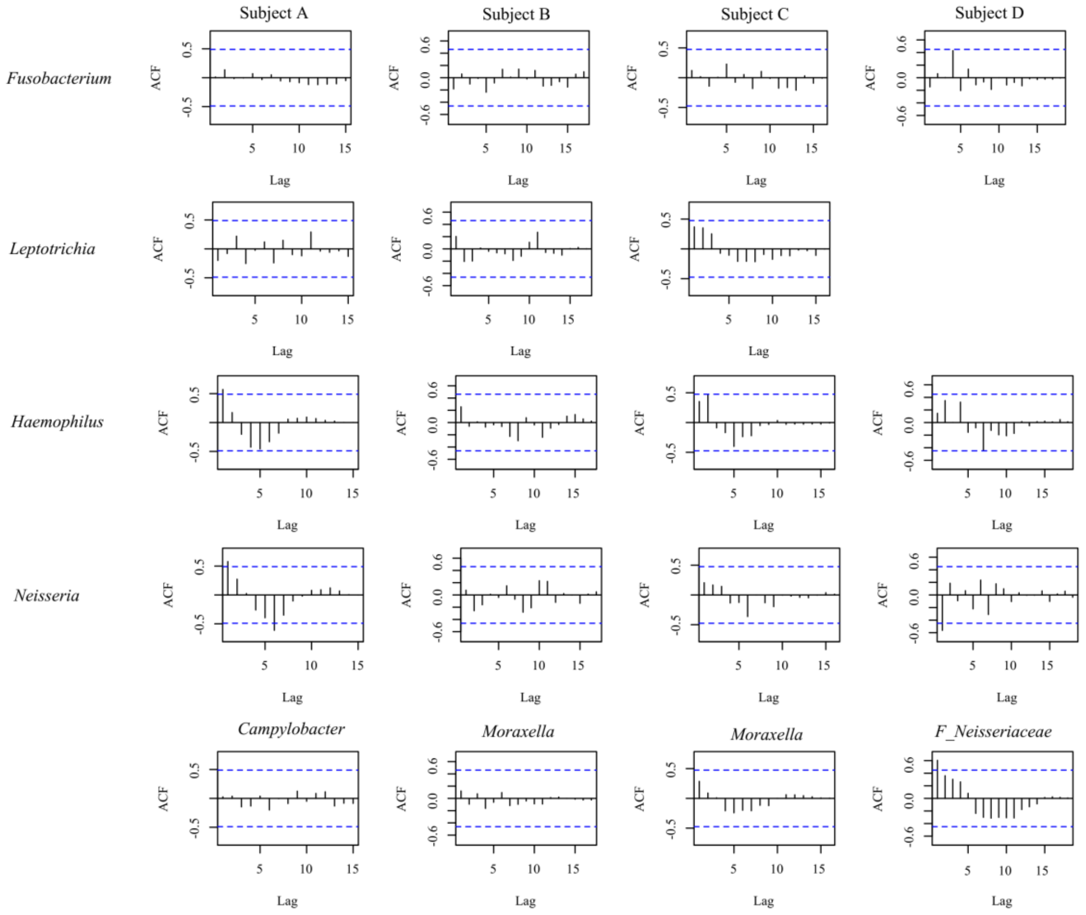

Fig S4: The sample autocorrelation function plots (ACF) of the highly abundant genera in crewmembers' saliva. Blue lines indicate upper and lower range of confidential region, sample autocorrelation that falls into the confidential region indicate stability of the genus. The high abundance genera are the genera of OTUs with the top 15 relative abundance in each crewmember. The results showed there was no consistent significant autocorrelation for crewmembers and the crewmembers' saliva microbiota variations at genus were stationary stochastic process.

Supplementary Fig. 5

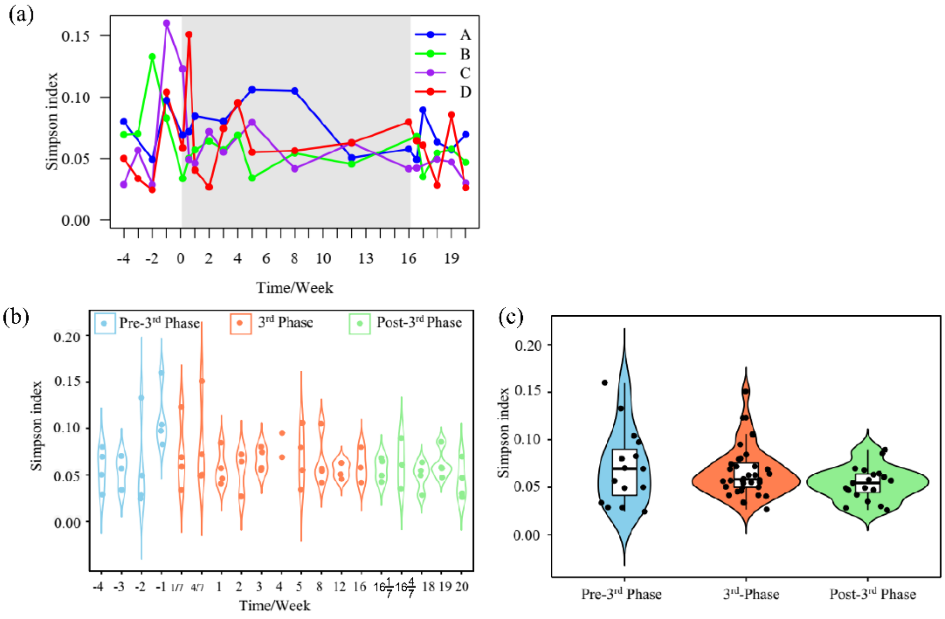

Fig S5: Changes in Simpson index of saliva microbiota over time. (a) Changes in Simpson index of crewmembers over time; (b-c) Violin maps of Simpson index at different time points and stages.Differences between groups were compared using the Kruskal-Wallis rank sum test. (Figure S3 (b): KW = 16.9120, $P = 0.5292$; Figure S3 (c): KW = 2.3652, $P = 0.3065$).

Supplementary Fig. 6

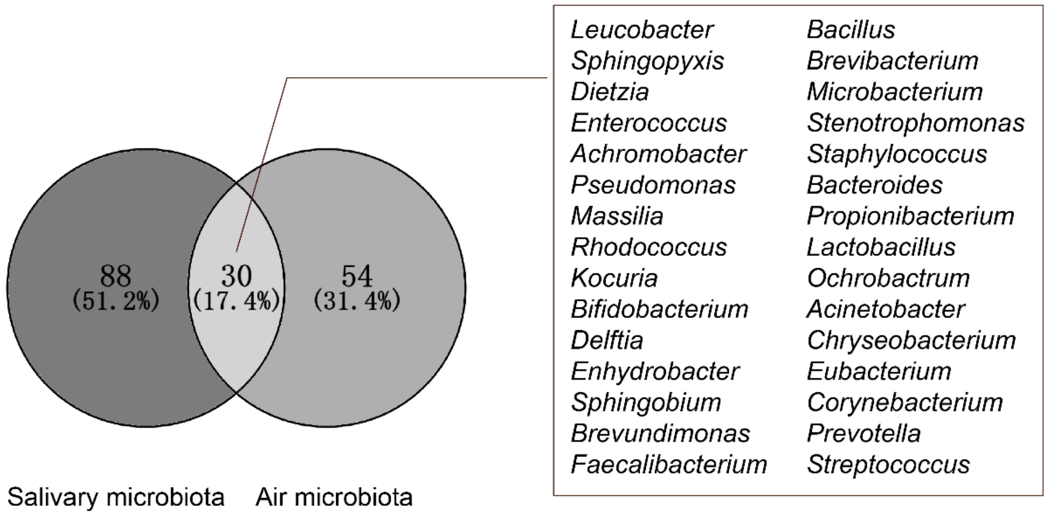

Supplementary Fig S6: VENN diagram of all detected bacterial genera in the air and salivary samples in the LP1.

Supplementary Fig. 7

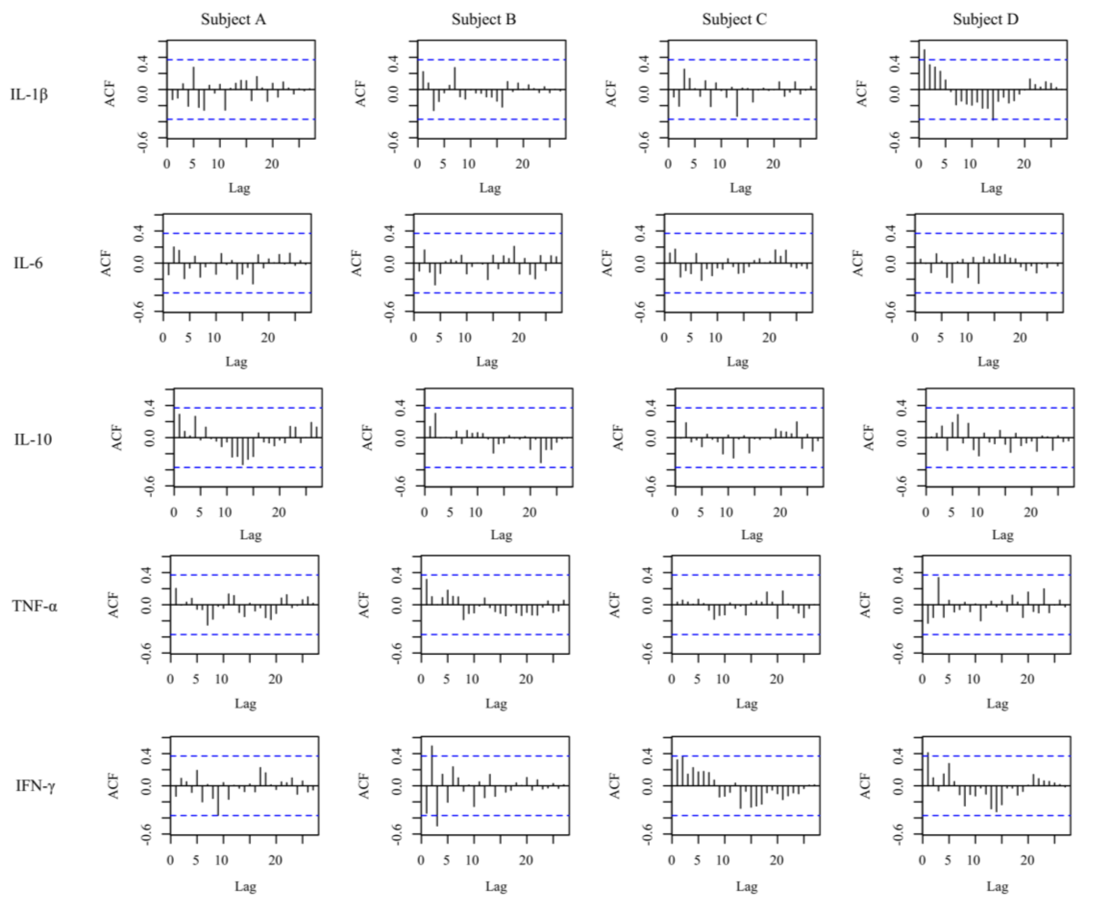

Fig S7: The sample autocorrelation function plots (ACF) of crewmembers' salivary cytokines. Blue lines indicate upper and lower range of confidential region, sample autocorrelation that falls into the confidential region indicate stability of the salivary cytokine. The results showed there was no consistent significant autocorrelation for crewmembers and the crewmembers' salivary cytokines variations were stationary stochastic process.
